# Nrf2 signaling increases expression of ATP-binding cassette subfamily C mRNA transcripts at the blood–brain barrier following hypoxia-reoxygenation stress

**DOI:** 10.1186/s12987-017-0055-4

**Published:** 2017-03-16

**Authors:** Kathryn Ibbotson, Joshua Yell, Patrick T. Ronaldson

**Affiliations:** 1 Department of Pharmacology and Toxicology, College of Pharmacy, University of Arizona, 1295 N. Martin Avenue, P.O. Box 210202, Tucson, 85721 AZ USA; 2 Department of Pharmacology, College of Medicine, University of Arizona, 1501 N. Campbell Avenue, P.O. Box 245050, Tucson, AZ 85724-5050 USA

**Keywords:** Blood–brain barrier, Endothelial cell, Hypoxia, Multidrug resistance proteins, Nrf2 signaling, Transporters

## Abstract

**Background:**

Strategies to maintain BBB integrity in diseases with a hypoxia/reoxygenation (H/R) component involve preventing glutathione (GSH) loss from endothelial cells. GSH efflux transporters include multidrug resistance proteins (Mrps). Therefore, characterization of Mrp regulation at the BBB during H/R is required to advance these transporters as therapeutic targets. Our goal was to investigate, in vivo, regulation of *Abcc1*, *Abcc2*, and *Abcc4* mRNA expression (i.e., genes encoding Mrp isoforms that transport GSH) by nuclear factor E2-related factor (Nrf2) using a well-established H/R model.

**Methods:**

Female Sprague–Dawley rats (200–250 g) were subjected to normoxia (Nx, 21% O_2_, 60 min), hypoxia (Hx, 6% O_2_, 60 min) or H/R (6% O_2_, 60 min followed by 21% O_2_, 10 min, 30 min, or 1 h) or were treated with the Nrf2 activator sulforaphane (25 mg/kg, i.p.) for 3 h. *Abcc* mRNA expression in brain microvessels was determined using quantitative real-time PCR. Nrf2 signaling activation was examined using an electrophoretic mobility shift assay (EMSA) and chromatin immunoprecipitation (ChIP) respectively. Data were expressed as mean ± SD and analyzed via ANOVA followed by the post hoc Bonferroni *t* test.

**Results:**

We observed increased microvascular expression of *Abcc1, Abcc2,* and *Abcc4* mRNA following H/R treatment with reoxygenation times of 10 min, 30 min, and 1 h and in animals treated with sulforaphane. Using a biotinylated Nrf2 probe, we observed an upward band shift in brain microvessels isolated from H/R animals or animals administered sulforaphane. ChIP studies showed increased Nrf2 binding to antioxidant response elements on *Abcc1, Abcc2,* and *Abcc4* promoters following H/R or sulforaphane treatment, suggesting a role for Nrf2 signaling in *Abcc* gene regulation.

**Conclusions:**

Our data show increased *Abcc1, Abcc2,* and *Abcc4* mRNA expression at the BBB in response to H/R stress and that *Abcc* gene expression is regulated by Nrf2 signaling. Since these Mrp isoforms transport GSH, these results may point to endogenous transporters that can be targeted for BBB protection during H/R stress. Experiments are ongoing to examine functional implications of Nrf2-mediated increases in *Abcc* transcript expression. Such studies will determine utility of targeting Mrp isoforms for BBB protection in diseases with an H/R component.

## Background

Cerebral hypoxia and reoxygenation (H/R) is a component of various diseases including traumatic brain injury, cardiac arrest, and ischemic stroke [1]. Blood–brain barrier (BBB) integrity is modulated by production of reactive oxygen species (ROS) and subsequent oxidative stress in the setting of H/R [2]. For example, studies using bovine brain microvessel endothelial cells subjected to H/R stress reported discrete changes in tight junction protein localization that correlated with increased paracellular permeability to sucrose, a vascular marker that does not cross the intact BBB [3]. Similar observations have been reported in vivo where H/R induced disassembly of occludin oligomers in rat brain microvessels [4, 5]. Furthermore, studies in the same model system showed increased CNS accumulation of sucrose [5, 6] and dextrans [7], evidence indicating BBB dysfunction in response to H/R. Indeed, vascular changes induced by H/R can have deleterious consequences. Enhanced BBB permeabilization can lead to vasogenic edema and cause clinically significant increases in brain volume and intracranial pressure [8, 9]. Additionally, substances that are typically contained within the systemic circulation, including drugs, can leak into brain parenchyma and potentially cause neurotoxicity. Clearly, there is a critical need to preserve BBB integrity in diseases with an H/R component.

In order to develop therapeutic approaches that can confer BBB protection, it is essential to identify specific biological mechanisms that contribute to oxidative stress-induced damage of the brain microvasculature. Indeed, furthering our understanding of the endothelial cell antioxidant defense system will enable advancement of such pharmacological strategies. The endogenous antioxidant glutathione (GSH) is a vital component of this antioxidant defense system. In vivo studies have demonstrated that cerebral GSH levels are significantly decreased in response to reperfusion injury [10] and GSH depletion is associated with increased BBB permeability to both sucrose and sodium fluorescein [11]. Although this does not reflect large-scale BBB disruption, this leak is clinically significant by permitting increased paracellular transport of potentially toxic small molecules. Decreased GSH levels in response to H/R may involve membrane transport processes mediated by multidrug resistance proteins (Mrps). Mrps are members of the ATP-binding cassette (ABC) superfamily of efflux transporters, primarily transport organic anions and conjugated metabolites, and are encoded by genes from ABC subfamily C (i.e., *Abcc* genes) [2]. Both GSH and glutathione disulfide (GSSG) are known transport substrates for Mrp1, Mrp2, and Mrp4. For example, studies in primary cultures of rat astrocytes showed that GSH transport could be blocked using MK571, an established inhibitor of Mrp1 and Mrp2 [12–14]. Similarly, Mrp4 is also believed to be involved in transport of GSH in the brain [15]. Indeed, these observations point towards endogenous transporters that can be targeted to preserve endothelial GSH levels and provide BBB protection in the setting of H/R.

Effective targeting of Mrps to reduce GSH efflux and confer BBB protection requires identification and characterization of regulatory pathways that control expression of these transporters. One such pathway is signaling mediated by nuclear factor E2-related factor (Nrf2). Nrf2 is normally inactive in the cytoplasm and rapidly degraded when associated with Kelch-like ECH-associated protein 1 (Keap1). Under conditions of oxidative stress, Keap1 dissociates and allows Nrf2 to translocate to the nucleus and initiate transcription of genes containing an antioxidant response element (ARE) [16]. Nrf2 has been shown to induce expression of Mrp1, Mrp2, and Mrp4 and the genes that encodes these proteins (i.e., *Abcc1, Abcc2, Abcc4*) at the BBB as well as in other tissues [17–19]. At present, involvement of Nrf2 in regulating Mrp transporter expression at the BBB has not been evaluated under pathophysiological conditions.

In the present study, we show increased expression of *Abcc1, Abcc2, and Abcc4* mRNA transcripts in brain microvessels via Nrf2 signaling in the setting of H/R. Specifically, we show for the first time that H/R activates Nrf2 signaling at the BBB and that Nrf2 binds to an antioxidant response element in the promoter of all three genes that encode GSH transporting Mrp isoforms. These data provide critical information that can inform future studies aimed at targeting Mrp transporters to confer BBB protection in diseases with an H/R component.

## Methods

### Animals and treatments

All animal experiments were approved by the University of Arizona Institutional Animal Care and Use Committee and conform to National Institutes of Health guidelines. Female Sprague–Dawley rats (200–250 g) were obtained from Envigo (Denver, CO), housed under standard 12 h light/12 h dark conditions, and provided with food and water *ad libitum*. Female rats were purposely selected for this study in order to correlate our results with previous data on BBB transporter changes in the setting of H/R [20]. Animals were randomly assigned to treatment groups. Animals were subjected to hypoxic (Hx) insult (i.e., 6% O_2_) for 1 h as previously described [20]. Using blood-gas analysis, our laboratory has previously demonstrated that these conditions yield a severe, but recoverable, hypoxic insult [20]. Additionally, this model increases CNS expression of apoptotic markers [i.e., ratio of cleaved poly-ADP ribose polymerase (PARP) to uncleaved PARP] [20]. Rats were then euthanized or subjected to reoxygenation (i.e., 21% O_2_) for 10 min, 30 min, or 1 h. These time points were selected based upon our previous work examining BBB transport mechanisms in the setting of H/R [20]. As shown in Thompson et al. [20], discrete changes in BBB transporters can be observed during reoxygenation as early as 10 min following hypoxic insult. H/R animals were compared with animals subjected to Hx only and with normoxic (Nx) controls. A subset of animals was administered sulforaphane [25 mg/kg (1.0 ml/kg), i.p.; Sigma-Aldrich, St. Louis, MO], an established Nrf2 activator, dissolved in 0.9% saline as a positive control. Following Nx, Hx, H/R or 3 h sulforaphane treatment, animals were euthanized by decapitation and prepared for microvessel isolation.

### Microvessel isolation

Brain microvessels were harvested as previously described by our laboratory [20]. Following anesthesia with sodium pentobarbital [64.8 mg/ml (1.0 ml/kg) i.p.], rats were decapitated and brains were removed. Meninges and choroid plexus were excised and cerebral hemispheres were homogenized in 4 ml of microvessel isolation buffer (103 mM NaCl, 4.7 mM KCl, 2.5 mM CaCl_2_, 1.2 mM KH_2_PO_4_, 1.2 mM MgSO_4_, 15 mM HEPES, pH 7.4) containing protease inhibitor cocktail (Sigma-Aldrich). After homogenization, 8 ml of 26% dextran at 4 °C was added and homogenates were vortexed. Homogenates were then centrifuged (5600*g*; 4 °C) for 10 min and the supernatant was aspirated. Pellets were resuspended in 10 ml of microvessel isolation buffer and passed through a 70 μm filter (Becton–Dickinson, Franklin Lakes, NJ). Filtered homogenates were pelleted by centrifugation at 3000×*g* for 10 min. At this time, the supernatant was aspirated and the pellet, which is enriched in brain microvessels, was collected for use in further experiments.

### Quantitative real-time PCR analysis

Total RNA was extracted from brain microvessels isolated from rats subjected to Nx, Hx, and H/R using the Aurum Total RNA extraction kit (Bio-Rad, Hercules, CA). Extracted RNA was treated with amplification grade DNase I (Bio-Rad) to remove contaminating genomic DNA. The concentration of RNA in each sample was quantified spectrophotometrically by measuring UV absorbance at 260 nm. The iScript reverse transcriptase kit (Bio-Rad) was used to synthesize first-strand cDNA. Primer pairs were prepared by Integrated DNA Technologies (Coralville, IA) with sequences listed in Table 1. Each set of primers was designed with the use of Primer Express 3 software (Applied Biosystems) and validated for specificity and efficacy by using BioTaq universal rat normal tissue cDNA (BioTaq Inc., Gaithersburg, MD). Primer pairs were designed to be complementary to sequences located on two different exons separated by an intron in order to avoid amplification of genomic DNA. Quantitative PCR was performed using SYBR Green Master Mix (Bio-Rad) on a CFX96 Touch Real-Time PCR Detection System (Bio-Rad). The quantity of the target gene (i.e., *Abcc1, Abcc2, Abcc4*) was normalized to GAPDH using the comparative *C*T method (ΔΔ*C*T). Results were expressed as mean ± SD of at least three separate experiments.

**Table 1:** Quantitative real-time PCR primer sequences

PCR primer sequences		
Gene	Forward primer	Reverse primer
*Abcc1 (rat)*	5′-TGC-CAG-AGA-TCA-GTT-CAC-ACC-AAG-CC-3′	5′-ACC-ATC-CGG-ACG-CAG-TTT-GAA-GAC-AG-3′
*Abcc2 (rat)*	5′-GAA-GGC-ATT-GAC-CCT-ATC-T-3′	5′-CCA-CTG-AGA-ATC-TCA-TTC-ATG-3′
*Abcc4 (rat)*	5′-TGG-AAC-TTC-TGG-AGG-ACG-GGG-ATC-TG-3′	5′-CCC-CTT-CTG-CAC-CAT-TTC-CGG-ATC-TT-3′
*GAPDH (rat)*	5′-ATG-GCT-ACA-GCA-ACA-GGG-TGG–TGG-AC-3′	5′-ATG-GGG-TCT-GGG-ATG-GAA-TTG-TGA-GG-3′

### Electrophoretic mobility shift assay (EMSA)

EMSA was performed using the LightShift Chemiluminescent EMSA kit (Pierce Biotechnology, Rockford, IL, USA) according to manufacturer’s instructions. Briefly, nuclear protein extract was isolated from brain microvessels prepared from Nx, Hx, H/R, and sulforaphane treated rats. Complementary DNA oligonucleotides 5′-CGG TCA CCG TTA CTC AGC ACT TTG-3′ and 5′-CAA AGT GCT GAG TAA CGG TGA CCG-3′ (antioxidant response element recognition sequence highlighted) were purchased from Integrated DNA Technologies, end labeled with biotin, and annealed at 95 °C for 5 min. EMSA samples were prepared using 5 μg of nuclear extract in each Nrf2/ARE binding reaction. The binding reaction was incubated at room temperature for 15 min and DNA–protein complexes were resolved on a precast 6% native polyacrylamide gel in 0.5% TBE buffer. The gel was removed from the electrophoresis unit, blotted onto a nitrocellulose membrane, and incubated with streptavidin-horseradish peroxidase for 30 min. Membranes were developed using enhanced chemiluminescence. Experiments to determine specificity of EMSA reactions for the Nrf2/ARE complex were conducted by incubating binding reactions in the presence of a rabbit monoclonal anti-Nrf2 antibody (EP1808Y; 1/20 dilution; Abcam, Cambridge, MA). Control EMSA experiments were performed by adding an excess (200×) of unlabeled probe to binding reactions.

### Chromatin immunoprecipitation (ChIP)

ChIP was performed using the Imprint Chromatin Immunoprecipitation Kit (Sigma-Aldrich) according to manufacturer’s instructions. Briefly, microvessels were isolated from Nx, Hx, H/R, and sulforaphane treated rats and subsequently cross-linked in buffer containing 1% formaldehyde for 10 min at room temperature. Cross-linking was stopped by addition of glycine to a final concentration of 125 mM followed by centrifugation at 180×*g* for 5 min at room temperature. At this time, the microvessel pellet was resuspended in 50 μl of Nuclei Preparation Buffer and incubated on ice for 10 min. Following centrifugation at 180×*g* for 10 min at 4 °C, the nuclear pellet was resuspended in shearing buffer and incubated on ice for 10 min. Chromatin was sheared to 200–1000 bp by sonication on ice. Sonicated chromatin was diluted twofold in lysis buffer and 100 μl of diluted sample per immunoprecipitation reaction was used. Each sample was added to individual wells of a 96-well assay plate where each well contained 1 μg of specific rabbit monoclonal anti-Nrf2 antibody (EP1808Y) that has been previously validated in ChIP assays [21]. Assay plates were incubated for 90 min at room temperature on an orbital shaker at 75 rpm. In parallel, a no-antibody sample was run as a negative control. At this time, 40 μl of DNA release buffer was added to each well and samples were incubated in a water bath at 65 °C for 15 min. Following this step, 40 μl of reversing solution was added to each well and samples were incubated in a water bath at 65 °C for 90 min. Washes and elutions were performed in accordance with manufacturer’s instructions for the Imprint ChIP assay kit. Eluted and input DNA samples were purified using a spin column to a final volume of 50 μl. Quantitative real-time PCR was performed using 2 μl of template DNA per 25 μl of polymerase chain reaction (PCR) amplification scale as described by Hoque and colleagues [22]. Quantification of Nrf2 occupancy to the ARE within *Abcc* gene promoter by SYBR green real-time PCR was performed using primer sets prepared by Integrated DNA Technologies (Table 2). All measurements were performed in triplicate and results were verified in three separate chromatin preparations.

**Table 2:** ChIP primer sequences

Primer sequences for ChIP		
Gene	Forward primer	Reverse primer
*Abcc1*	5′-GCT-GTG-TTA-CCA-GAA-CTG-CC-3′	5′-AGC-ACA-AGC-AGA-GTC-AGG-AT-5′
*Abcc2*	5′-CAG-GGC-TTT-GGA-GAA-GTG-ATA-3′	5′-GGA-AGC-AGA-TGT-TAA-GGA-GCA-A-3′
*Abcc4*	5′-CTT-GAG-GCT-GGG-AGT-TCT-AGG-G-3′	5′-ACT-GAC-AGA-GTG-GTG-TAG-CTG-GT-3′

### Statistical analysis

Data are reported as mean ± SD from at least three separate experiments where each treatment group consists of pooled microvessels from three individual animals (n = 3). This sample size is based upon the ability to detect a 35% difference between treatment groups with 20% variability. To determine statistical significance, a repeated measures ANOVA and post hoc multiple-comparison Bonferroni *t* test were used. A value of p < 0.05 was accepted as statistically significant.

## Results

### H/R increases expression of Abcc mRNA transcripts

**Fig. 1:**
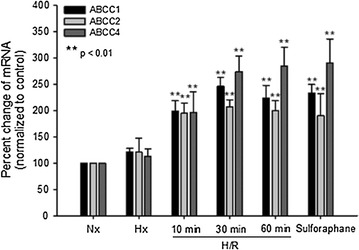
Increased mRNA expression of *Abcc1*, *Abcc2* and *Abcc4* at the BBB following H/R. Female Sprague–Dawley rats were subjected to H/R (Hx = 6% O_2_, 1 h; R = 21% O_2_ for 10 min, 30 min or 1 h), normoxia (Nx), hypoxia (Hx), or administered the Nrf2 activator sulforaphane for 3 h. Results are expressed as mean ± SD of three experiments, with each group consisting of pooled microvessels from three individual animals. *p < 0.01

In order to evaluate and quantitate mRNA expression of *Abcc* mRNA transcripts at the BBB in the setting of H/R, we performed quantitative PCR. After completion of these experiments, we observed increased expression of *Abcc1*, *Abcc2*, and *Abcc4* mRNA in rat brain microvessels following H/R treatment with reoxygenation times of 10 min, 30 min, and 1 h as compared to Nx controls or animals subjected to Hx insult only (Fig. 1). Expression of mRNA for all three genes was also increased in animals treated with the Nrf2 activator sulforaphane (25 mg/kg i.p.) for 3 h (Fig. 1). These studies demonstrate that H/R can increase expression of *Abcc* mRNA at the BBB.

### H/R induces nuclear translocation of Nrf2 in rat brain microvessels

**Fig. 2:**
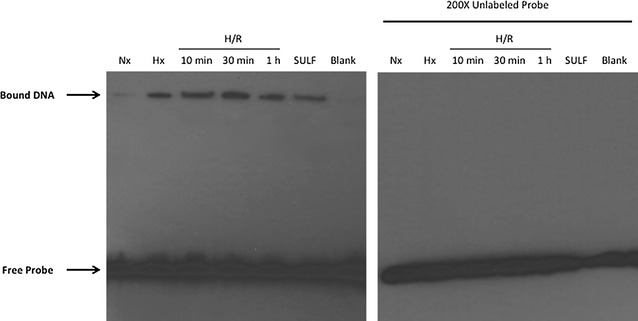
H/R induces nuclear translocation of Nrf2 in rat brain microvessels. EMSA experiments (*left-hand panel*) show band shift to a higher molecular weight and an increase in band intensity in brain microvessels from female Sprague–Dawley rats subjected to H/R (H = 6% O_2_, 1 h; R = 21% O_2_ for 10 min, 30 min or 1 h) and hypoxic (Hx) animals, as well as those treated with sulforaphane. The *right-hand panel* shows data from EMSA experiments conducted in the presence of an excess (×200) of unlabeled probe. Image depicts a representative blot from three separate experiments

**Fig. 3:**
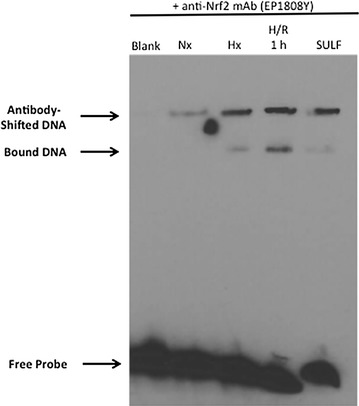
H/R induces nuclear translocation of Nrf2 in rat brain microvessels. EMSA experiments using a specific Nrf2 monoclonal antibody (EP1808Y) show a “supershift” of bands corresponding to the nuclear Nrf2/ARE complex in brain microvessels from female Sprague–Dawley rats subjected to H/R (H = 6% O_2_, 1 h; R = 21% O_2_ for 10 min, 30 min or 1 h) and hypoxic (Hx) animals, as well as those treated with sulforaphane. Image depicts a representative blot from three separate experiments

Since our qPCR data showed increased expression of *Abcc* genes at the BBB in response to H/R, we sought to identify a discrete molecular mechanism that is involved in induced expression of *Abcc* mRNA transcripts. We postulated that the Nrf2 pathway is one such mechanism. Therefore, we utilized an EMSA with a biotinylated probe containing the Nrf2 consensus binding sequence to demonstrate activation of Nrf2 signaling in rat brain microvessels following H/R. We observed a shift of the probe band to a higher molecular weight and an increase in intensity of the probe band in microvessels isolated from H/R animals or administered sulforaphane (25 mg/kg i.p.) for 3 h (Fig. 2). This shift was not observed when 200-fold excess unlabeled probe was added to EMSA reactions (Fig. 2). Incubation of EMSA reactions in the presence of the specific rabbit anti-Nrf2 monoclonal antibody EP1808Y caused an increase in the shift of the probe band, an observation that further indicates nuclear translation of Nrf2 under H/R conditions (Fig. 3). Taken together, these data provide evidence that H/R can activate Nrf2 signaling in brain microvessels.

### Nrf2 is involved in regulation of Abcc mRNA transcripts in rat brain microvessels following H/R

**Fig. 4:**
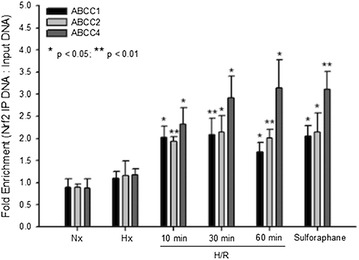
Involvement of Nrf2 in regulation of *Abcc* mRNA transcripts in rat brain microvessels following H/R. ChIP was performed on brain microvessels isolated from rats subjected to H/R (H = 6% O_2_, 1 h; R = 21% O_2_ for 10 min, 30 min or 1 h), Nx, Hx, or treated with sulforaphane for 3 h. Results are expressed as mean ± SD of three experiments, with each group consisting of pooled microvessels from three individual animals. *p < 0.05; **p < 0.01

Since our EMSA experiments demonstrated increased Nrf2 nuclear translocation in rat brain microvessels following H/R, we hypothesized that this pathway may be involved in transcriptional regulation of *Abcc* genes. To test this hypothesis, we used ChIP to study Nrf2 recruitment to promoter regions on Abcc genes that contain the Nrf2 consensus binding sequence [i.e., (a/g)TGA(C/T/G)nnnGC(a/g)] within the ARE. In H/R animals or in animals administered the Nrf2 activator sulforaphane, increased Nrf2 binding was determined for *Abcc1*, *Abcc2*, and *Abcc4* (Fig. 4). No difference in Nrf2 binding was observed in a non-specific region of the same promoter (data not shown). These data show that Nrf2 can bind to the promoter of Abcc genes in brain microvessels under H/R conditions, providing evidence for molecular regulation of drug efflux transporters (i.e., Mrps) at the BBB.

## Discussion

The mammalian Mrp family belongs to the ABCC group of proteins, which contains 13 members including one ion channel (i.e., CFTR), two surface receptors (i.e., SUR1 and 2) and a truncated protein that does not mediate transport (i.e., ABCC13) [2, 23]. Several functionally characterized Mrp isoforms have been localized to the mammalian BBB. These include Mrp1, Mrp2, Mrp4, Mrp5 and Mrp6 [24–30]. The presence of multiple Mrp isoforms at the BBB is a critical determinant in controlling delivery of therapeutic agents to the brain. Additionally, the ability of Mrp isoforms to actively efflux the endogenous antioxidant glutathione (GSH) has significant implications for diseases with an H/R component. GSH is responsible for maintenance of cellular redox balance and antioxidant defense in the brain. It has been previously demonstrated that various Mrps are upregulated in response to oxidative stress conditions, which leads to enhanced cellular efflux of GSH [14]. Increased functional expression of Mrp isoforms at the BBB could cause reduced endothelial cell concentrations of GSH, an alteration in cellular redox status, and increased potential for cell injury and death. Therefore, biological mechanisms that can modulate Mrp expression at the BBB in response to oxidative stress require further investigation.

A thorough understanding of signaling pathways involved in Mrp regulation in the setting of H/R will enable development of pharmacological approaches to target Mrp-mediated efflux (i.e., GSH transport) for the purpose of preventing BBB dysfunction. One intriguing pathway is signaling mediated by Nrf2, a sensor of oxidative stress [19, 31]. In the presence of ROS, the cytosolic Nrf2 repressor Keap1 undergoes structural alterations that cause dissociation from the Nrf2-Keap1 complex. This enables Nrf2 to translocate to the nucleus and induce transcription of genes that possess an antioxidant response element at their promoter [32, 33]. It has been demonstrated that activation of Nrf2 signaling induces expression of Mrp1, Mrp2, and Mrp4 [17–19, 32, 34, 35]. Our data expands upon these previous studies by showing Nrf2-mediated increases in mRNA transcript expression for *Abcc1,* Abcc2, and *Abcc4* in rat brain microvessels. We also show increased Nrf2 nuclear translocation in the setting of H/R and that Nrf2 binds to the ARE in the respective promoter for *Abcc1, Abcc2,* and *Abcc4. O*ur findings are novel and highly significant because we have shown, for the first time, that H/R-induced activation of Nrf2 leads to increased expression of mRNA transcripts for transporters endogenously expressed at the BBB. This is a rapid response, which may indicate that genes involved in the H/R stress response may be available for immediate activation in an effort to protect the vasculature from dysfunction and subsequent leak of circulating solutes. It is also intriguing that changes in *Abcc* mRNA transcript expression occur following H/R but are not apparent in the setting of hypoxia despite activation of Nrf2 signaling under both conditions. Such changes may be reflective of the dramatic increase in ROS production following H/R. For example, Fabian and Kent demonstrated increased production of superoxide anions by neutrophils following reperfusion, an event that can greatly exacerbate BBB dysfunction [8, 36]. Such dramatic increases in ROS production can certainly induce cellular changes independent of signaling pathways that are activated in response to hypoxia [37].

Recent evidence has shown that Nrf2 is a component of a complex signaling pathway, which involves additional factors for promoter activation and subsequent modulation of transport mechanisms at the BBB. For example, sulforaphane-induced increases in ABC transporter functional expression at the BBB can be abolished using pifithrin, an inhibitor of p53 signaling, or in p53 null mice [19]. In contrast, nutlin-3, a p53 activator, increased P-gp transport activity in mouse brain capillaries [19]. Of particular note, this study also demonstrated that pharmacological inhibitors of p38 MAPK signaling (i.e., SB203580) and nuclear factor-κB (NF-κB) signaling (i.e., *N*4-[2-(4-phenoxyphenyl)ethyl]-4,6-quinazolinediamine, SN50) blocked effects of sulforaphane and nutlin-3 on P-gp activity [19]. Taken together, the work of Wang and colleagues suggests that effects of Nrf2 signaling on ABC transporters at the BBB requires involvement of p53, p38 MAPK, and NF-κB signaling.

An emerging concept is that Nrf2 acts as a double-edged sword [33]. Activation of Nrf2 signaling at the BBB is generally considered to be protective owing to its activation of cytoprotective pathways; pre- and post-treatment administration of Nrf2 activators confer BBB protection in animal models of stroke and traumatic brain injury [38–40]. A subset of Nrf2 target genes are involved in the synthesis and metabolism of GSH, including GCLC and GCLM (subunits of glutamate–cysteine ligase), glutathione peroxidase, and glutathione reductase [33]. Indeed, increased expression of GSH synthetic genes can lead to increased cellular production of this critical antioxidant. However, oxidative stress increases the functional expression of Mrp1 [14], and oxidative stress induced by metals or H_2_O_2_ has been previously shown to increase Mrp1-mediated export of GSH and GSSG [13, 41–43]. Upregulation of Mrp isoforms in glial cells may have neuroprotective effects in the setting of oxidative stress through release of GSH into brain parenchyma where it can be readily accessed by neurons [41, 44]. However, an alteration in the balance of Mrp isoforms via activation of Nrf2 signaling may have considerably different effects than in brain parenchyma. Indeed, efflux of GSH by Mrp isoforms expressed at the abluminal membrane of the BBB may provide some neuroprotection; however, increased GSH efflux due to enhanced Mrp-mediated transport can adversely affect redox balance and antioxidant defense at the brain microvascular endothelium and contribute to barrier dysfunction in the setting of H/R. This indicates that studies designed to develop pharmacological approaches based on targeting Mrp isoforms at the BBB must consider both neuroprotective and vascular protective effects associated with these transporters. Additionally, increased functional expression of Mrp isoforms at the BBB can negatively affect endothelial cell inflammation and repair pathways. Endogenous mediators involved in such pathways include leukotriene C4, a known Mrp1/Mrp2 substrate [45, 46], and prostaglandin E_2_ [47].

In order to fully comprehend the implications of Nrf2-mediated upregulation of *Abcc* gene expression at the BBB, future studies must be undertaken to assess Mrp localization in the brain microvasculature. Expression and localization of Mrp isoforms at the BBB is species-dependent and remains highly controversial [48, 49]. Localization of Mrp1 is thought to be at the abluminal plasma membrane in brain microvascular endothelial cells in rodents, but at the luminal membrane in humans [50, 51]. Mrp4 has been detected on the luminal surface of the BBB in rat; however, abluminal expression has not been confirmed [50, 51]. Based on qPCR and proteomic analysis, Mrp4 is the most abundant of the three GSH-transporting isoforms in human brain microvessels [29, 50]. Mrp2 is likely localized to the luminal aspect of the BBB, but several studies have failed to detect Mrp2 at the protein level [49, 51]. This may be due to low basal expression of Mrp2, which may be increased in response to cellular stressors such as oxidative stress [28, 52]. In mice, there are notable differences in Mrp expression between strains and between vessels of different diameters. For example, FVB mice appear to lack Mrp2 in brain vessels, but it is present in C57BL/6 and Swiss mice [53]. This same study also showed that Mrp1is most abundant in vessels 20–50 μm in diameter [53]. Rigorous assessment of Mrp isoform localization will undoubtedly inform the development of therapeutic strategies to protect the BBB in diseases with an H/R component. Furthermore, these studies should include both male and female experimental animals in order to determine differences in Mrp localization based on sex.

## Conclusion

**Fig. 5:**
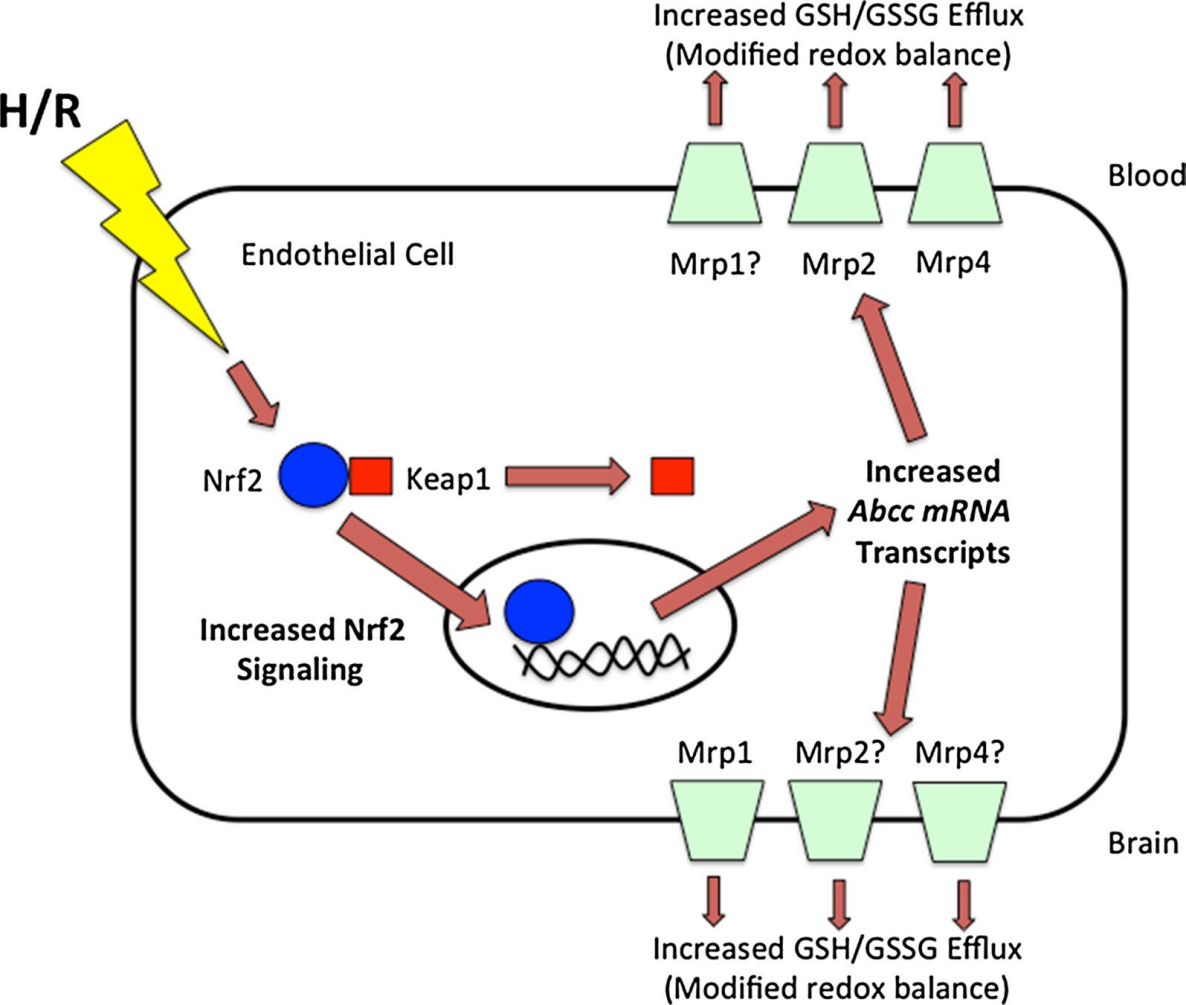
Prevention of BBB dysfunction by targeting Mrp isoforms at the BBB. Results from our present study demonstrate increased mRNA expression of *Abcc1*, *Abcc2*, and *Abcc4* at the BBB following an H/R insult. Furthermore, H/R stress is known to suppress GSH levels and increase GSSG concentrations in the brain. We propose that changes in GSH/GSSG transport occur during H/R as a result of altered functional expression of at least one Mrp isoform. Since Nrf2, a ROS sensitive transcription factor, is known to regulate Mrps, we hypothesize that this pathway is a critical regulatory mechanism for Mrps at the BBB. Our present data show involvement of Nrf2 signalling in regulation of *Abcc* mRNA transcript expression in rat brain microvessels following H/R. Future studies are ongoing in our laboratory to determine the functional implications of this observation, particularly with respect to GSH transport and redox balance at the BBB. Mrp isoforms where BBB localization has not been confirmed are indicated by (*question mark*)

Our data show increased *Abcc1, Abcc2,* and *Abcc4* mRNA expression at the BBB in response to H/R stress and that *Abcc* gene expression is regulated by Nrf2 signaling (Fig. 5). This is the first time that Nrf2 signaling has been shown to modulate *Abcc* genes at the brain microvasculature in the setting of H/R stress. Since Mrp1, Mrp2, and Mrp4 transport GSH, these results have considerable pharmacological implications as they point to endogenous transporters that can be targeted for development of novel therapeutic strategies to confer BBB protection. Furthermore, our in vivo H/R treatment does not induce necrotic damage to the endothelium, thus enabling us to study a dynamically regulated and recoverable BBB. Such a model can inform novel strategies to target the penumbra in ischemic stroke, which is subject to hypoxic insult but can be potentially rescued using pharmacological interventions. Future studies are ongoing in our laboratory to examine functional implications of Nrf2-mediated increases in *Abcc* transcript expression, particularly with respect to Mrp protein expression and brain-to-blood transport of GSH, in order to rigorously examine the utility of Mrp isoforms as a therapeutic target in diseases with an H/R component.

## Abbreviations

ABC: ATP-binding cassette
ARE: antioxidant response element
BBB: blood–brain barrier
ChIP: chromatin immunoprecipitation
GSH: glutathione
GSSG: glutathione disulfide
H/R: hypoxia-reoxygenation
Keap1: Kelch-like ECH-associated protein 1
Mrp: multidrug resistance protein
Nrf2: nuclear factor E2-related factor
PARP: poly-ADP ribose polymerase
ROS: reactive oxygen species
